# The actin-bundling protein Fascin is overexpressed in inflammatory bowel disease and may be important in tissue repair

**DOI:** 10.1186/1471-230X-11-14

**Published:** 2011-02-23

**Authors:** David Qualtrough, Katie Smallwood, David Littlejohns, Massimo Pignatelli

**Affiliations:** 1 School of Cellular and Molecular Medicine, Medical Sciences Building, University of Bristol, University Walk, Bristol, BS8 1TD, UK

## Abstract

**Background:**

Fascin is associated with increased cell motility in colorectal tumours but is absent from the normal colonic epithelium. We examined the expression of fascin in inflammatory bowel disease (IBD) and its location at regions undergoing restitution and regeneration. Tissue repair is essential for disease remission and we sought to determine the effects of therapeutic modalities on fascin expression and function using an *in vitro *model.

**Methods:**

Immunohistochemistry was performed on colonic tissue from IBD patients to determine changes in fascin expression and distribution. A human colorectal epithelial cell line was treated with 5-aminosalicylate (a common treatment for IBD), or sodium butyrate to determine the effect on fascin expression and cell motility.

**Results:**

Fascin overexpression was observed in both ulcerative colitis and Crohn's colitis and expression correlated with disease severity. Immunoreactivity was more intense and widespread in Crohn's compared to ulcerative colitis. Interestingly, highly expressing foci were consistently observed at the edges of ulcers where flattened, motile epithelial cells are actively involved in restitution, and also in areas of mucosal regeneration.

5-aminosalicylate reduced fascin expression in colorectal epithelial cells and inhibited their motility. Conversely, sodium butyrate increased fascin expression and stimulated cell motility in the same cells.

**Conclusions:**

Our data shows that fascin is overexpressed in inflammatory bowel disease and its location is indicative of a role in tissue repair. Our *in vitro *studies show that different therapeutic modalities may have converse effects on fascin expression and may have significant consequences for disease remission and the clinical management of IBD.

## Background

Ulcerative colitis (UC) and Crohn's disease (CD) are forms of inflammatory bowel disease (IBD) which affect an estimated 1.4 million people in the USA and 2.2 million across Europe. UC exclusively affects the large intestine, whereas CD affects the colon in 60% of cases (known as Crohn's colitis), but can also involve other parts of the GI tract [1]. These common, chronic and debilitating conditions remain incurable, with their aetiology and pathogenesis not clearly understood. Long-term illness greatly increases the risk of colorectal cancer, which causes approximately 15% of all IBD patient deaths [2]. The onset of cancer in IBD patients is sudden, rapid, and highly aggressive, with a poor prognosis. The occurrence of dysplasia in IBD is widely accepted to be pre-malignant, but the likelihood of progression to cancer is difficult to predict [3]. Even the distinction between low grade- and high-grade dysplasia provides little indication of disease outcome [2] and there is, therefore, an urgent need for biomarkers to predict neoplasia in IBD.

Both UC and CD are characterised by mucosal infiltration of inflammatory cells and frequent epithelial damage. This damage can result in destruction of the mucosa and a breach in the barrier that this tissue provides against the luminal milieu. Complete remission of IBD requires both a reduction in inflammation and repair of the damaged epithelium. Inadequate or incomplete repair can result in the formation of a 'leaky barrier' which can in turn perpetuate a vicious cycle of chronic inflammation [4]. The process of 'healing' areas of the mucosa devastated by inflammation is widely accepted to be a two-stage process comprising 'restitution' and 'regeneration' [5]. Restitution is characterised by flattening and spreading of the epithelium at the margins of the ulcer, with these cells migrating across the denuded sub-mucosa to cover the damaged area. Following restitution, the regeneration programme requires widespread epithelial cell proliferation and formation of characteristic glandular structures of the intestinal crypts. Although several cytokines and growth factors have been implicated in the restoration of epithelium following injury, the cellular processes underpinning this mechanism remain poorly understood [5].

Current therapy for IBD, particularly UC, centres on the long-term administration of the non-steroidal anti-inflammatory drug (NSAID) 5-amino salicylate (5-ASA). Shown to be effective in controlling intestinal inflammation in the majority of patients, 5-ASA also reduces colorectal cancer risk in patients with IBD [6,7].

Other workers in the field have proposed sodium butyrate, a fermentation product of dietary fibre, as a potential therapy for IBD. Trials using butyrate irrigation led to symptomatic amelioration in UC patients [8,9]. Luminal levels of butyrate may be modulated through the dietary intake of fibre and are found in the millimolar range [10].

Among the most pressing current issues in the clinical management of IBD are a need to further our understanding of intestinal wound healing and to understand the effects of therapeutic modalities on tissue repair as this is crucial for disease remission.

Fascin (fascin-1) is a 55kDa actin-bundling protein which localises to the core actin bundles of spikes and filopodia at the leading edge of migratory cells. It is known to be overexpressed in various cancers and has been shown to increase motility in cells from several different tissues [11]. Work in this laboratory has shown fascin to be completely absent from the normal colorectal epithelium but widespread in colorectal tumours [11,12]. Fascin expression has been shown to correlate with an increased risk of malignant progression and with a poorer disease prognosis making it a potential biomarker in colorectal neoplasia [12,13].

As yet, there are no published reports of fascin expression in IBD, but it is our hypothesis that fascin will be involved in tissue repair in IBD. The changes in motile behaviour demonstrated by tumour cells mimic those occurring during tissue repair and require dynamic rearrangements in the actin cytoskeleton, governed by actin-binding proteins such as fascin.

The first aim of this study was to determine the expression of fascin in clinical samples of IBD with particular attention to areas of restitution and regeneration. Samples of resection margins from patients undergoing surgery for diverticulitis were included as these show low-grade mucosal inflammatory infiltration.

Secondly, in order to elucidate the potential modulation of fascin by therapeutic intervention and the subsequent consequences for epithelial repair, we studied the effects of 5-ASA and sodium butyrate on fascin expression and cell motility using an *in vitro *cell line model.

We now show, for the first time, that fascin is overexpressed in IBD and expression is associated with regions of active mucosal repair. Furthermore, therapeutic modalities for IBD can affect fascin expression and colonic epithelial cell motility.

## Methods

### Ethical considerations

Ethical approval was obtained from the North Somerset & South Bristol Research Ethics Committee (REC reference 04/Q2003/49). All tissue samples were obtained from the files of the Department of Histopathology, Bristol Royal Infirmary. These samples were anonymised by a third party and the investigators had no access to patient information.

### Immunohistochemistry

A total of 41 surgical specimens of resected colorectal tissue from IBD patients were immunohistochemically stained for fascin as described previously [11,12]. We also examined samples of normal colorectal mucosa and 11 samples of resection margins from patients undergoing surgery for diverticulitis as these display low grade mucosal inflammation.

Fascin was detected using a mouse monoclonal antibody (Dako-Cytomation, Glostrup, Denmark) as previously described [11,12]. Negative controls had no primary antibody applied.

Stained samples were scored by two independent observers in terms of the proportion of epithelial cells staining positive (1 for <20%, 2 for >20%) and also the intensity of epithelial staining relative to that observed in adjacent endothelial cells (0,1,2 for negative, weak, moderate/strong, respectively) which act as an internal positive control [11,12]. From the anonymised histology reports, disease activity was scored 0, 1, or 2 for quiescent, low/moderate, or severe activity, respectively.

### Statistical analysis

The immunohistochemistry scores were analysed for specific correlations using Kendall's tau B analysis as previously described [12]. Correlations were checked between proportion and intensity of fascin immunoreactivity, disease activity, and the presence of dysplasia. These analyses yield a value between zero and one (one being the strongest possible correlation and zero indicating no correlation at all) and a p-value to indicate the significance of the correlation. A p-value of less than 0.05 is considered statistically significant (*, p = < 0.05; **, p = < 0.01; ***, p = < 0.001).

### Cell Lines and treatments

The HT29 cell line was originally derived from a sporadic colonic adenocarcinoma and was maintained in culture in DMEM supplemented with 10% FBS [14].

For treatments, 1 × 10^6 ^cells were seeded per 25 cm^2 ^culture flask in triplicate for each dose and control, and the cells allowed to recover for 48 hours prior to treatment. 5-ASA (Sigma) was dissolved in DMEM supplemented with 2% FBS to produce a 50 mM solution and the pH adjusted to 7.4. This stock solution was diluted further in DMEM (2% FBS) for cell treatments. The 5-ASA was made up fresh immediately prior to each experiment and protected from light throughout the procedure. Sodium butyrate (Sigma) treatments were carried out as described previously [15]. Apoptosis was assessed as previously described [15]. The level of apoptosis in cultured colon cells can be assessed by measuring the proportion of cells that detach from the flask and float in the medium [15 and references therein] Apoptosis was confirmed in these floating cells by morphology (following acridine orange staining). Acridine orange staining was performed as previously described with at least 300 cells scored for each sample. The proportion of cells exhibiting apoptotic morphology was >90% in the floating cell population, and this proportion remained constant following treatment. Therefore, the proportion of cells floating in the medium can be used as a measure of the extent of apoptosis in the culture. Western blotting analysis was performed on attached cells-thereby precluding apoptotic cells from this measure of fascin expression.

### Western Blot Analysis

Cell lysates of 2 × 10^6 ^cells were prepared for western blotting as described previously [16]. A mouse monoclonal antibody raised against fascin was obtained from Dako (Dako-Cytomation, Carpinteria, CA). Blots were subsequently probed with anti α-tubulin (Sigma, UK) to show equal sample loading.

### Cell motility assays

Cell migration assays were carried out using a transwell filter migration assay as previously described [12]. The lower chamber was filled with Calcium Free-DMEM supplemented with 5% FBS to act as an attractant. Following a 24 hour incubation and staining with haemotoxylin, cells on the lower filter surface were considered migratory and counted in 10 fields at ×20 magnification.

## Results

### Fascin expression is expressed in inflamed colonic epithelium

**Figure 1:**
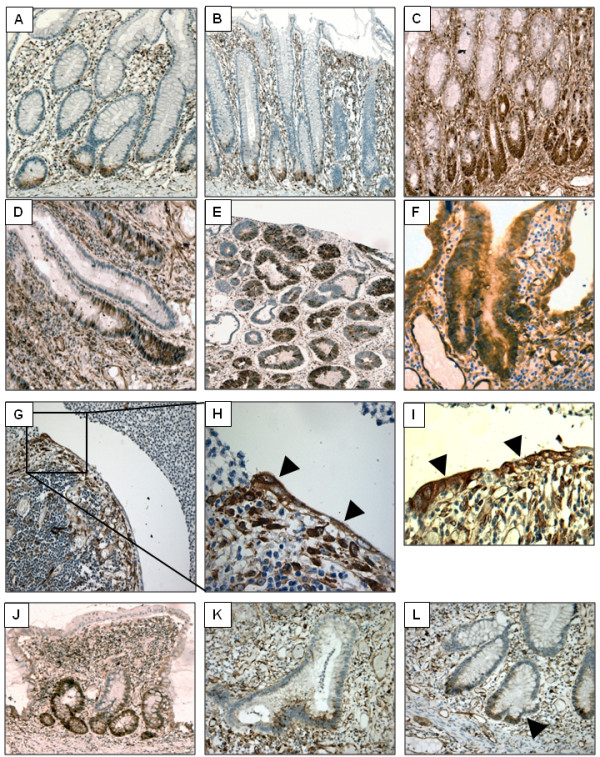
**Fascin is overexpressed in inflammatory bowel disease and in colonic epithelium actively undergoing restitution and regeneration**. Fascin immunoreactivity was detected in specimens of human colorectal mucosa by immunoperoxidase staining. A: Diverticulitis resection margin showing positive staining in the crypt base - 200 × magnification. B: Ulcerative colitis showing positive staining in the base of the elongated crypts - 100 ×. C: Crohn's colitis showing strong positive staining toward the base of the elongated crypts - 100 ×. D: Crohn's colitis showing patchy fascin immunoreactivity throughout the gland - 200 ×. E: Ulcerative colitis with low-grade dysplasia showing strong and widespread fascin expression - 100 ×. F: Ulcerative colitis with high-grade dysplasia showing strong and widespread fascin expression - 200 ×. G: Ulcerative colitis showing fascin staining in the flattened epithelial cells undertaking restitution - 200 ×. H: enlargement of an area of panel G illustrating the fascin-positive epithelial cells (arrowheads). I: fascin positive epithelial cells undertaking restitution in a sample of Crohn's colitis. J: A regenerative polyp in a sample of ulcerative colitis showing fascin positivity in the newly forming crypts - 200 ×. K & L: Fascin expression in branching crypts (arrowheads) in regenerating ulcerative colitis tissue - 200 ×.

Previous studies have shown that fascin is not expressed in the epithelial cells of the normal colonic mucosa, but is broadly expressed in the endothelial cells, fibroblasts and infiltrating lymphocytes of the lamina propria and submucosa [11,12]. Our previous study of colorectal adenomas showed epithelial fascin expression focussed around the tumour stalk, provoking the hypothesis that fascin expression may be modulated by inflammatory mediators [12]. In order to test this hypothesis immunohistochemistry was first performed on 11 tissue samples of resection margins from patients undergoing surgery for diverticulitis as these samples show low level inflammation. In 10 out of the 11, epithelial fascin immunoreactivity was focally observed in epithelial cells at the very base of the colonic crypts (Figure 1A). This observation is the first time that fascin expression has been observed in non-neoplastic colorectal epithelium and led us to question the relationship between fascin and inflammatory conditions of the colon.

### Fascin is overexpressed in inflammatory bowel disease showing stronger and more widespread expression in Crohn's compared with ulcerative colitis

**Figure 2:**
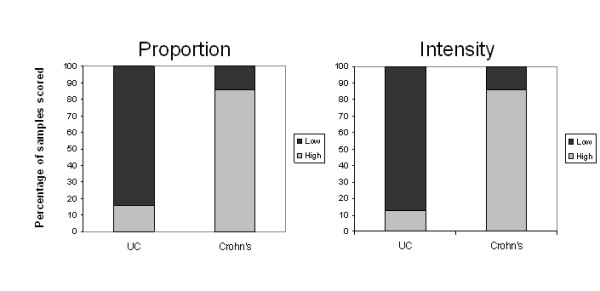
**Fascin expression is stronger and more widespread in Crohn's colitis compared with Ulcerative colitis**. A graphical summary of the immunohistochemical study of fascin expression in IBD. Samples were scored high or low for the proportion of epithelium staining positive (low = <20%; high = >20%), and the intensity of the epithelial fascin immunoreactivity. The graphs illustrate a significant increase in both the proportion (Ttest p = 0.0017) and intensity (Ttest p = 0.0013) of fascin immunoreactivity in Crohn's compared with ulcerative colitis samples.

Having shown that epithelial fascin expression was observed in the presence of low-level colonic inflammation, fascin immunohistochemistry was performed on 41 samples of resected colorectal mucosa from patients suffering from IBD. Fascin was widely overexpressed in the epithelium of IBD-involved tissue and representative results are shown in Figure 1. The data from the subsequent scoring of these samples is summarised in Figure 2. Strong fascin staining was frequently observed toward the crypt bases in the elongated crypts associated with IBD (Figure 1B and 1C) but also showed a more patchy distribution throughout the glands (Figure 1D).

Strikingly, following scoring of the immunostaining, it was found that both the proportion of positive epithelial cells and the intensity of fascin staining (relative to internal positive control) was significantly greater in samples of Crohn's compared with UC (Figure 2). Staining intensity also positively correlated with disease activity (as determined by the consulting pathologist) for both conditions (Tau B = 0.223, p = <0.05). The intensity of fascin stain observed in the sample was also found to correlate with the proportion of fascin-positive epithelium (Tau B = 0.621, p = < 0.001).

Previous study has shown that fascin is overexpressed in both the benign and malignant stages of colorectal neoplasia and is associated with malignant progression of these tumours [11,12]. Increased risk of colorectal cancer is an important clinical consideration in IBD, and malignant progression of areas of dysplasia remains highly unpredictable [2]. In this study, strong fascin expression was observed in IBD samples showing either low or high grade dysplasia (Figure 1E), or cancer. Both the intensity of epithelial fascin immunoreactivity (Tau B = 0.391, p = <0.05) and the proportion (Tau B = 0.406, p = < 0.01) of positive epithelial cells in the tissue significantly correlated with the presence of areas of dysplastic epithelium.

### Fascin is overexpressed in colonic epithelium actively undergoing restitution and regeneration

Inflammatory infiltration in IBD frequently results in complete destruction of the mucosal layer resulting in areas of ulceration. Repair of these gaps in the epithelial barrier occurs as a two-stage process, termed restitution and regeneration [5]. In the sample group used, all of the 11 sections showing signs of active restitution stained positive for fascin in the flattened epithelial cells moving to cover the denuded area (Figure 1G, H).

Fascin staining was also observed in the newly formed immature crypts of regenerative polyps (Figure 1J) and in epithelial glands undergoing crypt fission - a relatively common observation in IBD tissue undertaking regeneration (Figure 1K). Taken together, these data suggest that fascin could play an important role in IBD and could be vital for disease remission through modulating mucosal repair.

### 5-aminosalicylate reduces fascin expression and cell motility whereas sodium butyrate has a converse effect

We have established above that fascin is overexpressed in IBD, and that expression is associated with areas undertaking repair. As fascin is known to promote cell motility in neoplastic colorectal epithelial cells [12], and motility is a key factor in epithelial restitution, we aimed to determine the effect of therapeutic modalities used in the treatment of IBD on fascin expression and cell motility *in vitro*.

5-aminosalicylate (5-ASA) is widely used in the clinical management of IBD, whereas the short chain fatty acid sodium butyrate, a luminal fermentation product of dietary fibre, has been proposed for use in IBD treatment [6,9].

**Figure 3:**
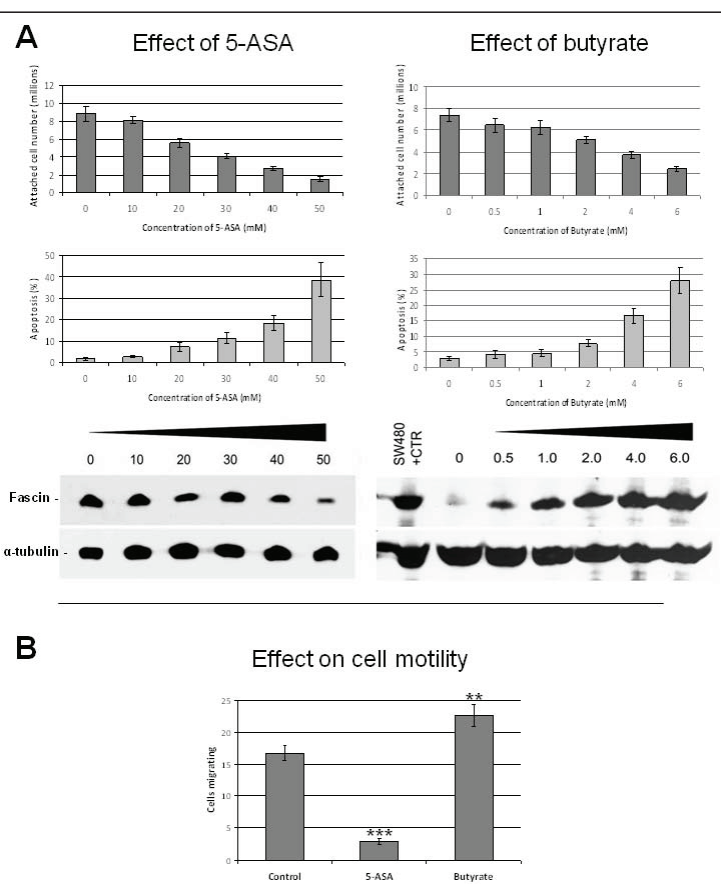
**5-aminosalicylate reduces fascin expression and cell motility in colorectal epithelial cells, whereas sodium butyrate has a converse effect**. **A: **HT29 cells were treated with a range of doses of either 5-ASA or butyrate as indicated. Attached cell yields and percentage of apoptotic cells were determined and these data represent the mean of three independent experiments performed in triplicate ±SEM. Also shown are the subsequent western blot analyses of fascin expression. These blots are representative of three independent experiments and were re-probed for α-tubulin to show even sample loading. SW480 lysate was included as a positive control having been shown previously to express relatively high levels of fascin [Qualtrough *et al*., 2009]. **B: **The effect of 10 mM 5-ASA or 1 mM butyrate treatment on cell motility was determined on HT29 cells as measured by Boyden chamber assay using 5%FBS as an attractant (**, p = <0.01; ***, p = <0.001). Three independent experiments were carried out in triplicate and the data are expressed as the mean ± S.E.M.

In order to determine the effect of 5-ASA or butyrate on fascin expression we treated HT29 colorectal epithelial cells with a range of doses. Following 48 hours of treatment, the cells were counted and samples prepared in order to assay fascin expression by western blotting (Figure 3A). Both 5-ASA and butyrate reduced attached cell yield and induced apoptosis in the HT29 cells as described previously [15]. Apoptotic cells were not included in the analysis of fascin expression.

5-ASA treatment caused a decrease in fascin expression compared with control, whereas butyrate strongly enhanced fascin expression in a dose-dependent manner (Figure 3A). Promoter reporter assays using the full length fascin promoter [17] showed that regulation occurred, in both cases, at the transcriptional level (data not shown).

In order to determine whether 5-ASA and butyrate affect cell motility in this *in vitro *model, cells were seeded on collagen-coated transwell filters as previously described [12] and simultaneously treated with either 5-ASA or butyrate at doses which were shown not to significantly induce apoptosis (10 mM and 1 mM, respectively-Figure 3A). Motile cells were counted 24 hours later (Figure 3B). 5-ASA significantly reduced cell motility in HT29 cells whereas, conversely, butyrate stimulated cell migration.

## Discussion

We show here, for the first time, that the actin bundling protein fascin is overexpressed in IBD, and may play an important role in tissue repair following inflammatory damage. Our *in vitro *findings suggest that therapeutic approaches for IBD could influence fascin expression, subsequently producing unwanted side-effects on tissue repair and thereby influencing achievement of remission.

We have shown previously that fascin is absent from the normal colorectal epithelium but overexpressed in both benign and malignant tumours where it is associated with enhanced cell motility [11,12]. Our previous findings in colorectal adenomas, where the tumour stalk frequently showed the strongest fascin immunoreactivity, suggest fascin regulation occurs through epithelial-mesenchymal crosstalk, potentially through inflammatory mediators [12]. This notion of inflammatory induction of fascin expression in the epithelium is supported here by the widespread expression of fascin in tissue resected from patients with IBD. A positive correlation was observed between the disease activity recorded by the pathologist and the intensity of fascin immunoreactivity further supporting the idea of an inflammation-mediated regulation of fascin expression. Also in keeping with these results is the observation of epithelial fascin expression at the crypt base in tissue from patients undergoing surgery for diverticulitis, which display low-grade inflammation.

The increased proportion and intensity of fascin staining observed in samples of Crohn's compared with UC raises questions about the fundamental difference between these classifications of IBD. As it was only the epithelial immunoreactivity that was scored, this difference cannot be attributed to the ability of Crohn's colitis to affect deeper layers of the bowel wall than UC, but rather resides in the mechanism of fascin regulation.

IBD carries an increased risk of colorectal cancer, resulting in the deaths of 15% of IBD patients [2]. Malignant progression is highly unpredictable and therefore the identification of prognostic biomarkers of tumourigenic potential is of key importance [2]. In this study we showed that both the proportion and intensity of epithelial fascin stain significantly correlated with the presence of dysplasia or cancer. Further study, of a larger sample group with long-term clinical follow-up, will be necessary to confirm the validity of fascin as a biomarker for tumorigenesis in IBD. The role of fascin in the malignant progression of sporadic colorectal tumours [12] does suggest that fascin merits further investigation in the neoplastic transformation of IBD.

One of the most intriguing observations in our study of fascin expression in IBD was the focal expression of fascin in regions demonstrating active tissue repair. Fascin expression was found in all areas of restitution observed, and also in regenerative polyps and branching crypts, suggesting a role throughout the various stages of the repair process. Our previous work has shown that fascin can promote motility in benign, as well as malignant colorectal epithelial cells [12], and it would have been reasonable to hypothesise that fascin would be expressed in the motile cells undertaking restitution. Furthermore, fascin has been shown to modulate cell adhesion and could therefore also function in the dynamic adhesive changes required for both restitution and crypt fission [11]. These data suggest that regulation of fascin could be important in achieving and maintaining remission in patients with IBD.

One of the most broadly used treatments for the maintenance of remission in IBD is 5-ASA and previous work has shown this drug to induce apoptosis in HT29 cells in a dose-dependent manner [18]. We show here for the first time that treatment of the colorectal epithelial cell line HT29 with this drug led to a decrease in fascin expression and a retardation of cellular motility.

Long term use of 5-ASA in patients with IBD is known to reduce cancer risk [19,20]. We postulate based on our findings here that this chemopreventive effect may, in part, be explained by the down regulation of fascin expression-with fascin being known to be involved in malignant progression of the colorectal epithelium [12]. This repression of cellular motility represents a key anti-cancer effect of 5-ASA treatment.

Conversely, considering the potential role of fascin in tissue repair, this would suggest that 5-ASA could hinder wound repair and thus impede remission in patients with active disease. The published evidence is somewhat conflicting as there are reports to suggest that NSAIDs can both impair wound healing [21] and that 5-ASA can promote intestinal repair [22]. However, the possibility exists that 5-ASA could have some detrimental effects in IBD patients in addition to its obvious benefits. This paradox in the effect of 5-ASA, and also the role of fascin, could have important consequences for the clinical management of IBD. To this end, our findings suggest that increased luminal levels of butyrate could complement 5-ASA in promoting remission whilst maintaining potent chemopreventive effects against tumorigenesis.

Previous work has suggested that sodium butyrate could have beneficial effects for IBD sufferers [8,9]. This fermentation product of dietary fibre is known to be present in the colonic lumen in the millimolar range and levels can be modulated by intake of suitable dietary substrate [10]. We, and others, have previously shown that butyrate has potent chemopreventive effects against colorectal tumorigenesis through the modulation of differentiation and apoptosis in colorectal epithelial cells [15,23,24].

We show here that butyrate upregulates fascin expression and significantly stimulates colorectal epithelial cell motility. These data suggest that butyrate will aid colonic tissue repair and therefore speed remission in IBD. This short chain fatty acid has already been proposed to promote wound healing in the small intestine [25]. Although our data suggest that fascin may be involved in tumorigenesis, butyrate has been shown to elicit potent anti-tumour effects. We propose that the combinatorial use of butyrate, or dietary modulation of its levels, could favourably complement 5-ASA use in the clinical management of IBD.

## Conclusions

We have shown for the first time that fascin is important in the pathogenesis and remission of IBD and could have important implications for its clinical management as fascin can potentially be manipulated using existing therapeutic approaches and modulation of diet.

## Competing interests

The authors declare that they have no competing interests.

## Authors' contributions

DQ: Conceived, designed, and coordinated the study and acquired the necessary funding; supervised the laboratory projects of KS & DL; carried out additional immunohistochemistry and all subsequent analyses; carried out some of the *in vitro *experiments; drafted the manuscript. KS & DL: Carried out the immunohistochemistry and some of the *in vitro *studies. MP: Contributed to the design and coordination of the study and aided with manuscript preparation. All authors read and approved the final manuscript.

## Pre-publication history

The pre-publication history for this paper can be accessed here:

http://www.biomedcentral.com/1471-230X/11/14/prepub
